# The Difference in Repeatability of Automated Superficial Retinal Vessel Density according to the Measurement Area Using OCT Angiography

**DOI:** 10.1155/2020/5686894

**Published:** 2020-04-17

**Authors:** Hyung Bin Lim, Tae Seen Kang, Yeo Kyoung Won, Jung Yeul Kim

**Affiliations:** ^1^Department of Ophthalmology, Chungnam National University College of Medicine, Daejeon, Republic of Korea; ^2^Department of Ophthalmology, Gyeongsang National University Changwon Hospital, Changwon, Republic of Korea

## Abstract

**Purpose:**

To evaluate the difference in the repeatability of automated superficial retinal vessel density and foveal avascular zone (FAZ) metrics according to the measurement area of optical coherence tomography angiography (OCTA).

**Methods:**

A total of 127 normal eyes from 127 healthy subjects were included. Macular angiography images were acquired from all subjects using the Zeiss Cirrus 5000 with AngioPlex^™^ OCTA software. Scans of 3 × 3 mm and 6 × 6 mm were each performed twice in a randomly arranged sequence. Vessel density (VD), perfusion density (PD), and FAZ metrics of the superficial capillary plexus were calculated automatically for all scans, and the repeatabilities for both scan patterns were assessed based on intraclass correlation (ICC), coefficient of variation (CV), and coefficient of repeatability (CR) parameters. The average measured values in the two scan patterns were also compared.

**Results:**

VD was significantly greater in the 3 × 3 mm scan than in the 6 × 6 mm scan according to all parameters, whereas PD was significantly less in the 3 × 3 mm scan than in the 6 × 6 mm scan. The ICCs for VDs in the central fovea were 0.826 and 0.741 for the 3 × 3 and 6 × 6 mm scans, respectively, and the CVs were 8.00% and 12.75%. For PDs, the ICCs were 0.839 and 0.762 and the CVs were 9.32% and 14.90%. The FAZ metrics in the 3 × 3 mm scan showed good repeatability with an ICC >0.75 and a CV <10.0%. However, all ICCs for the 6 × 6 mm scans were <0.75, and the CVs were all >10%.

**Conclusions:**

The 6 × 6 mm macular angiography scans resulted in lower repeatabilities than the 3 × 3 mm scans according to all OCTA parameters, particularly in the central fovea and FAZ metrics. The 3 × 3 mm scan was more suitable than the 6 × 6 mm scan for analyzing macular microvascular density and FAZ metrics.

## 1. Introduction

Recently introduced optical coherence tomography angiography (OCTA) is a novel imaging technique that provides fast and noninvasive assessment of the retinal and choroidal capillary network [1]. OCTA can differentiate and visualize the microvasculature of various retinal and choroidal layers at different depths, which is difficult to obtain using conventional fluorescein or indocyanine green angiography. The AngioPlex™ from the Zeiss Cirrus 5000 (Carl Zeiss Meditec, Dublin, CA, USA) is a commercially available OCTA instrument. It quantifies the vascular density (VD), perfusion density (PD), and foveal avascular zone (FAZ) in the superficial capillary plexus (SCP).

Changes in retinal VD and FAZ are potential biomarkers for macular ischemia in diabetic retinopathy and other retinal vascular diseases [2, 3], which may be useful for monitoring and detecting retinal vascular diseases, including diabetic retinopathy and retinal vascular occlusion. Recent studies have reported the normal ranges of VDs [4–6] and FAZs [7] measured with OCTA. However, various factors can influence OCTA measurements, and consideration of these factors is needed for correct OCTA data analyses.

Several studies have analyzed OCTA measurements and reported good repeatability and reproducibility [8–10]. Many physicians currently use OCTA for the diagnosis of glaucoma as well as retinal and neuro-ophthalmic diseases. However, various factors, including the OCTA instrument, scan area, and image quality, can affect the measured values, and no consensus concerning these factors has been established. For microvascular image acquisition, the operator can change the scan area. If the measurement area is changed, the sampling density per unit area will be changed [11], which may affect the OCTA measurement. Hence, this study examined which of the two commonly used angiography scans, 3 × 3 mm or 6 × 6 mm, is more reliable with the Cirrus HD-OCT 5000.

## 2. Materials and Methods

### 2.1. Subjects

This was a prospective and cohort study. This study initially included 151 eyes with no clinical evidence of ophthalmic disease from 151 healthy individuals who visited the retina clinic of the Chungnam National University Hospital (Daejeon, Republic of Korea). The study protocol was approved by our Institutional Review Board and adhered to the tenets of the Declaration of Helsinki. All included subjects met the eligibility criteria and provided written informed consent to participate.

All subjects underwent comprehensive ophthalmic examinations including a slit-lamp examination, best-corrected visual acuity (BCVA) tests, intraocular pressure (IOP) measurements, dilated fundus examination, fundus photography, and an axial length measurement using the IOLMaster^®^ (Carl Zeiss Meditec, Jena, Germany). The inclusion criteria included an age of 20–79 years, BCVA >20/25 (Snellen), a spherical equivalent within ±6 diopters (*D*), a high-quality fundus image, the absence of glaucomatous and other optic neuropathies, the absence of retinal nerve fiber layer defects using fundus photography, and no previous intraocular surgeries or procedures such as intraocular injection or retinal laser photocoagulation. Exclusion criteria included the presence of diabetes or hypertension, a history of retinal neuro-ophthalmic disease and glaucoma, a history of ocular trauma, BCVA <20/25, IOP >21 mmHg, spherical equivalent >+6.0 *D* or <−6.0 *D*, axial length ≥26.0 mm, OCTA scan signal strength <9, and the presence of segmentation errors or motion artifacts in the angiography scan. Finally, 127 eyes from 127 healthy subjects were included in this study.

### 2.2. OCTA

**Figure 1:**
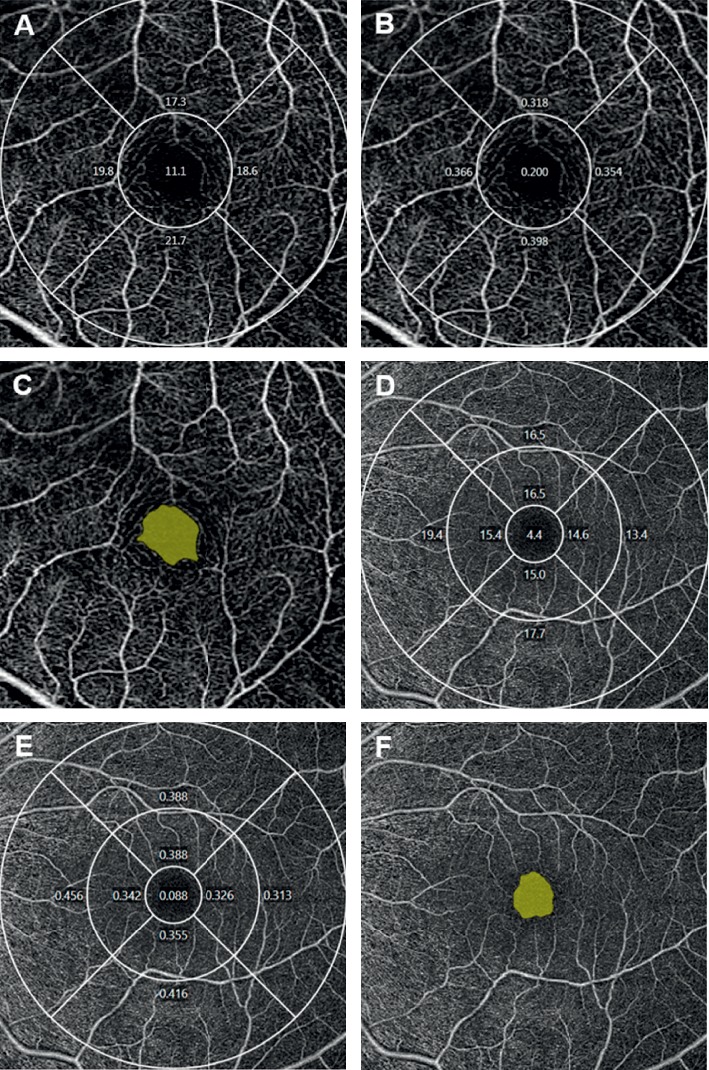
Representative optical coherence tomography angiography (OCTA) images acquired from a 3 × 3 mm scan (upper row) and 6 × 6 mm scan (lower row). A vessel density map of the superficial capillary plexus (SCP) according to the Early Treatment of Diabetic Retinopathy Study (ETDRS) subfields (a, d). A perfusion density map of the SCP according to the ETDRS subfields (b, e). The automatically detected foveal avascular zone (c, d).

The Zeiss Cirrus HD-OCT model 5000 with AngioPlex™ (Carl Zeiss Meditec) was used to acquire the microvasculature images of macular areas. All eyes underwent macular angiography imaging with two 3 × 3 mm scans and two 6 × 6 mm scans in a randomly arranged sequence, with a total of four consecutive scans under pupil dilation (Figure 1). All subjects were given at least 5-minute break between each scan, and “Track to prior scan” mode was not used during the study. This instrument operated at a central wavelength of 840 nm and a speed of 68,000 A-scans per second. In the 3 × 3 scan pattern, there were 245 A-scans in each B-scan along the horizontal and vertical dimensions. The 6 × 6 mm scan pattern contained 350 A-scans in each B-scan along the horizontal and vertical dimensions [11]. The optical microangiography-complex (OMAG^c^) algorithm analyzed the changes in complex signals (both intensity and phase changes contained within the sequential B-scans performed at the same position) [12, 13] and then produced en face microvascular images. The vascular images of the SCP, which spanned from the internal limiting membrane to the inner plexiform layer, and deep capillary plexus (DCP), which extended from the inner nuclear layer to the outer plexiform layer, were displayed automatically. The AngioPlex™ incorporates the FastTrac™ retinal-tracking technology to minimize motion artifacts.

All scans were analyzed using the Cirrus OCTA software (AngioPlex™, version 10.0). The measurement area of the 6 × 6 mm scan was divided into nine subfields according to the Early Treatment of Diabetic Retinopathy Study (ETDRS), and the 3 × 3 scan was composed of a 1 mm center and four quadrant sectors (superior, inferior, nasal, and temporal) that were identical to the inner circles of the ETDRS subfields (Figure 1). VD (defined as the total length of perfused vasculature per unit area in a region of measurement) and PD (defined as the total area of perfused vasculature per unit area in a region of measurement) of each subfield were measured automatically. Area, perimeter, and circularity (defined as 4πA/P, where A was the area and P was the perimeter) [14] of the FAZ were also measured. All OCTA scans were performed by the same experienced examiner, and all scans were reviewed individually by two investigators (HBL and TSK) for quality evaluation (i.e., loss of fixations, segmentation errors, and motion artifacts), and substandard scans were excluded.

### 2.3. Repeatability Assessment

Repeatability was assessed based on intraclass correlations (ICCs), coefficients of variation (CVs), and coefficients of repeatability (CRs). The within-subject standard deviation (Sw) was calculated as the square root of the within-subject variance [15]. CV was calculated as (Sw/average of the measurements) × 100, and values < 10% indicated good reproducibility. CR was defined as 1.96 × $\sqrt{2}$ × Sw [16]. If the measurements were normally distributed, the absolute difference between the two measurements differed by no more than CR for 95% of the measurements. The ICC is a statistical parameter that summarizes the reproducibility of a given group of subjects. It is based on variance component analyses and indicates the variance attributable to real differences between subjects as a part of the total variation [17]. The ICCs were interpreted as poor (ICC < 0.40), fair (0.40 ≤ ICC < 0.60), good (0.60 ≤ ICC < 0.75), and excellent (ICC ≥ 0.75) [18].

### 2.4. Statistical Analyses

To analyze the differences in repeatabilities according to angiography scan area, the ICC, CV, and CR for two 3 × 3 mm scans and two 6 × 6 mm scans were calculated and compared to each other. Bland–Altman plots were also used in the analyses [16]. The average values of the two scans using the VD, PD, and FAZ from each type of scan were compared using the paired *t*-test. This comparative analysis was performed in the area where the two scan patterns matched, such as the microvascular density of the fovea and inner subfields, and the FAZ metrics.

All statistical analyses were performed using SPSS statistical software for Windows, version 18.0 (SPSS, Chicago, IL, USA) and MedCalc, version 14.8 (MedCalc, Ostend, Belgium). Snellen BCVA results were converted into the logarithm of the minimum angle of resolution value (logMAR). Continuous variables are presented as the mean ± standard deviation. Differences were considered significant at *p* < 0.05.

## 3. Results

### 3.1. Demographics

This study included 68 males and 59 females, and the mean age was 48.3 ± 16.0 years, and the mean BCVA was −0.03 ± 0.08 logMAR. Clinical examination revealed no abnormal findings in any subjects. The baseline characteristics of the study population are shown in Table 1.

### 3.2. Comparison of OCTA Measurements between the 3 × 3 mm and 6 × 6 mm Scans

The mean VD of the central fovea using 3 × 3 mm scans was 10.53 ± 8.37 mm^−1^, which is significantly higher than that of the 6 × 6 mm scans (9.93 ± 6.53 mm^−1^; *p* < 0.001; Table 2). The measured values of the ETDRS of all inner subfields in the 3 × 3 mm scans were significantly higher than those of the 6 × 6 mm scans (all, *p* < 0.001; see Supplementary material 1). In PD analyses, in contrast to the VD, all areas of the center and all inner subfields showed high measurements in the 6 × 6 mm scans, which were statistically significant (all, *p* < 0.001). There were no significant differences between scan types for the FAZ areas (0.245 ± 0.115 vs. 0.280 ± 0.120; *p*=0.484; Table 2). However, the FAZ perimeter was significantly higher in the 3 × 3 mm scans (2.269 ± 0.564 vs. 2.176 ± 0.581, *p*=0.018) and the circularity for the 6 × 6 mm scans (0.652 ± 0.088 vs. 0.704 ± 0.098; *p* < 0.001), respectively.

### 3.3. Repeatability of OCTA Measurements according to Angiography Scan Size

In the VD and PD repeatability analyses, the ICC values for the 3 × 3 mm scans were >0.75 and the CVs were <10% in all areas; however, the 6 × 6 mm scans showed ICCs <0.75 in all inner subfields for the VD, and the inner superior, inner inferior, and inner temporal areas for the PD. The CVs for the 6 × 6 mm scans in all subfields were <10%, except the fovea (12.75% for the VD and 14.90% for the PD; Table 3 and Supplementary material 2). In addition, in all matching areas in both scan types, the ICC, CV, and CR of the 3 × 3 mm scans were better than those of the 6 × 6 mm scans. The FAZ of the 3 × 3 mm scans, similar to the VD and PD, showed a high ICC, and low CV and CR values in the area, perimeter, and circularity, respectively (Table 3). All CV values of the 6 × 6 mm scans were >10%, and the ICC of the FAZ area, perimeter, and circularity were 0.695, 0.694, and 0.503, respectively.

**Figure 2:**
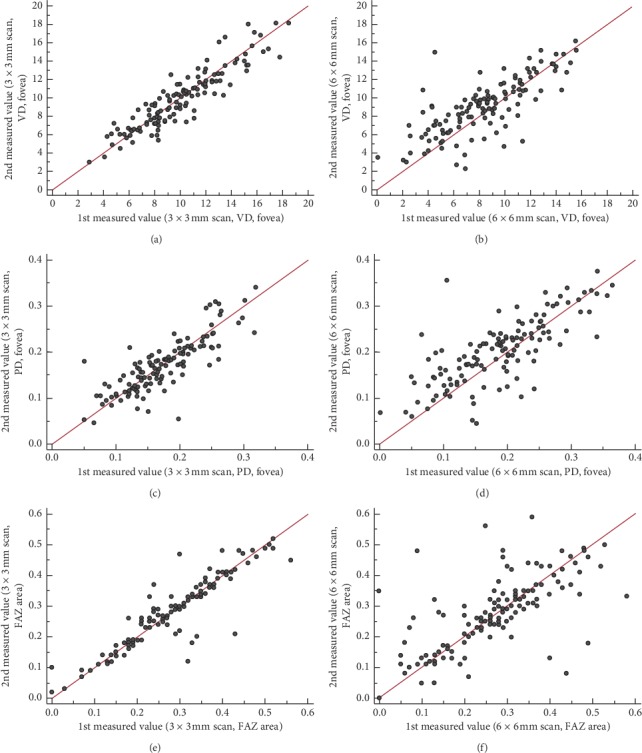
Scatter plots for vessel density (a, b), perfusion density (c, d), and foveal avascular zone area (e, f) measured automatically with a 3 × 3 mm scan (left column) and a 6 × 6 mm scan (right column). The *x*-axis and *y*-axis in all plots denote the first and second measured values, respectively.

**Figure 3:**
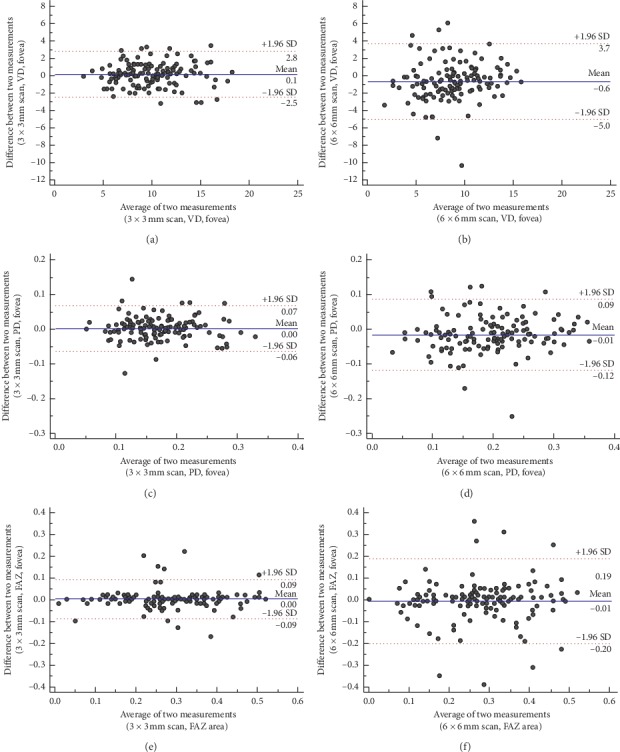
Bland–Altman plots showing the level of agreement between 3 × 3 mm (left column) and 6 × 6 mm scans (right column) for vascular density (a, b) and perfusion density (c, d) in the fovea and foveal avascular zone (e, f). The solid line denotes the mean difference, and the dashed lines denote the 95% confidence limits of agreement.

Scatter plots for the VD and PD in the fovea and FAZs showed a higher agreement of the two measurements for the 3 × 3 mm scans compared to the 6 × 6 mm scans (Figure 2). These trends were also confirmed in Bland–Altman plots, with a lower absolute difference between the two measurements and narrower 95% confidence lines (Figure 3).

## 4. Discussion

Because of the advantages of the OCTA-derived automated retinal vascular metrics, the use of OCTA in clinical practice continues to expand. Many recent studies have reported the use of OCTA for glaucoma and various retinal diseases. These studies have reported the VD and FAZ metrics in each disease. Assessment of repeatability is important for the reliability of acquired quantitative metrics of the retinal microvasculature during disease management and clinical trials.

In the present study, we performed two 3 × 3 mm scans and two 6 × 6 mm scans, a total of four scans in a random sequence with at least 5-minute intervals, and “Track to prior scan” mode was not used during the study to analyze repeatability. Finally, we found that both 3 × 3 mm and 6 × 6 mm scans using the AngioPlex™ software of the Cirrus HD-OCT model 5000 showed good repeatability in most measurements. A previous study of healthy subjects using the AngioVue^™^ (Optovue, Fremont, CA, USA) reported that the intraobserver ICCs were 0.64–0.93 [7], similar to the present study. Another study of the repeatability of the automated average VD using the Nidek RS-3000 Advance device (Nidek, Fremont, CA, USA) showed good repeatability with an ICC of 0.9 and CV of 5.2% [8]. Microvascular image acquisition in these studies used 3 × 3 mm scans. Lei et al. [19] reported high repeatabilities of 3 × 3 mm and 6 × 6 mm scans using the Cirrus HD-5000 AngioPlex™. However, there were no analyses of the fovea and FAZ. Guo et al. [9] reported good repeatability in the FAZ area using the Zeiss Cirrus HD-OCT model 5000. The CVs from two observers were 2.9% and 3.4%, and the ICCs were 0.997 and 0.996. Although the instrument was the same as that used in our study, the FAZ was measured manually by ImageJ software (National Institutes of Health, Bethesda, MD, USA).

In the present study, lower repeatabilities for all microvascular metrics were obtained using 6 × 6 mm scans compared to 3 × 3 mm scans. Using the 3 × 3 mm scans, the ICC for VD and PD ranged from 0.752 to 0.892, and the CV ranged from 3.41 to 8.00%. The ICC and CV for the 6 × 6 mm scans of the inner subfields ranged from 0.604 to 0.855 and from 5.66 to 12.75%, respectively. This trend was observed not only for VD and PD, but also for the FAZ metrics. The FAZ, perimeter, and circularity of the 3 × 3 mm scans showed a good ICC of >0.75, with a CV <10%, which indicates high repeatability. In the 6 × 6 mm scans, however, all CVs exceeded 10% and the ICC value of circularity was 0.503, the lowest value among all OCTA measurements. Scatter plots and Bland–Altman plots confirmed the better repeatability of the two 3 × 3 mm scans. These differences between the two scan patterns probably resulted from the following reasons. First, the 3 × 3 mm scan had better resolution than the 6 × 6 mm scan. Its scan interval was 12.2 *µ*m, compared to 17.1 *µ*m for the 6 × 6 mm scan [11]. This difference in scan density probably resulted in better repeatability for all OCTA measurements using the 3 × 3 mm scan. In addition, the 3 × 3 mm scan measured approximately 60,000 points, while the 6 × 6 mm scan measured approximately 120,000 points, taking twice as much time as the 3 × 3 mm scan, which might have caused increased motion artifacts.

Repeatabilities have shown different patterns according to the ETDRS subfield. The CV and CR for the central fovea were higher compared to other areas, particularly when using 6 × 6 mm scans. You et al. [20] reported that the intravisit CV value of the fovea was 4.9%, which was higher than that of other areas. They suggested that the lower repeatability in the central fovea might be related to the FAZ. In the central fovea, there are fewer blood vessels than in other subfields due to the FAZ, and mild changes in measurements can cause substantial variation. Repeatabilities in the outer subfields were higher than those of the inner subfields using 6 × 6 mm scans; the inner averages of the ICC, CV, and CR were 0.734, 5.66, and 2.47, respectively, and the outer averages of repeatability indicators were 0.828, 3.29, and 1.55, respectively. It is not clear why this difference occurred. We presumed that outer areas contained more large vessels that branched from the retinal vessel arcade, and the inner area could be affected by the FAZ. Additional studies are needed to further characterize these findings.

Munk et al. [21] compared four OCTA instruments including the Cirrus HD-OCT model 5000 and concluded that a direct comparison of OCTA metrics was difficult because each instrument had different analysis algorithms and scan resolutions. In addition, they also suggested that discrepancies could occur using different scan sizes because of different image resolutions and acquisition times. In the present study, we found that VD values were significantly higher using the 3 × 3 mm scans and the PD was higher using the 6 × 6 mm scans. In addition, there were significant differences between the two patterns for the FAZ perimeter and circularity, but not for the FAZ area. These discrepancies in microvascular densities and FAZ metrics may have resulted from the better scan resolution using the 3 × 3 mm scans. By contrast, the higher PD using the 6 × 6 mm scans might have resulted from the lower scan resolution and higher FAZ circularity in the 6 × 6 mm scan because of the inverse relationship with the perimeter.

We also evaluated the CR, which was the smallest real difference. The CR is a useful index that quantifies the absolute measurement error in reliability. The CRs of the VD and PD of the fovea were 2.37 mm^−1^ and 0.05 for the 3 × 3 mm scans, respectively, and 2.70 mm^−1^ and 0.08 for the 6 × 6 mm scans, respectively. In the FAZ, the CR of the 3 × 3 mm and 6 × 6 mm scans were 0.06 mm^2^ and 0.13 mm^2^, respectively, showing that any changes in microvascular metrics within the CRs were all within the smallest real difference and were nonsignificant. These findings must be considered when analyzing longitudinal changes in microvascular metrics.

This study had some limitations. We analyzed only the VD, PD, and FAZ in the SCP. Our OCTA software provided automated OCTA metrics only for the SCP, and not for the DCP. Due to projection artifacts, there may still be limitations regarding the analyses of retinal microvascular metrics in the choroid or deep retinal layers. Second, because this study was conducted on subjects with no ophthalmic disorders, our results cannot be applied to patients with retinal or other ocular diseases because of poor fixation or segmentation errors, so the CR values from this study may not be applicable to these patients.

In conclusion, better repeatability for all indicators was obtained using 3 × 3 mm macular angiography scans compared to 6 × 6 mm scans. In particular, VD and PD measurements in the central fovea and FAZ metrics showed lower repeatabilities in the 6 × 6 mm scans. Based on the overall results of this study, 3 × 3 mm scans are more suitable than 6 × 6 mm scans for quantitatively analyzing macular microvascular metrics and the FAZ.

## Figures and Tables

**Table 1:** Demographics.

Eyes (no.)	127
Age (mean ± SD, years)	48.3 ± 16.0
Sex (male/female)	68 : 59
BCVA (mean ± SD, logMAR)	−0.03 ± 0.08
Spherical equivalent (mean ± SD, diopters)	−1.32 ± 2.71
Intraocular pressure (mean ± SD, mmHg)	15.5 ± 3.0
Axial length (mean ± SD, mm)	24.2 ± 1.2

SD, standard deviation; BCVA, best-corrected visual acuity; logMAR, logarithm of the minimum angle of resolution.

**Table 2:** Comparisons of the vascular density, perfusion density, and foveal avascular zone metrics between 3 × 3 mm and 6 × 6 mm scans.

	Mean values of two 3 × 3 mm scans	Mean values of two 6 × 6 mm scans	*p* value
Vessel density			
Total area (mean ± SD, mm^−1^)	20.77 ± 1.80	18.53 ± 0.60	
Center (mean ± SD, mm^−1^)	10.53 ± 8.37	9.93 ± 6.53	**<0.001**
Inner average (mean ± SD, mm^−1^)	22.06 ± 1.75	18.65 ± 0.92	**<0.001**
Perfusion density			
Total area (mean ± SD)	0.359 ± 0.013	0.434 ± 0.015	
Center (mean ± SD)	0.171 ± 0.017	0.196 ± 0.028	**<0.001**
Inner average (mean ± SD)	0.382 ± 0.013	0.422 ± 0.022	**<0.001**
Foveal avascular area			
Area (mean ± SD, mm^2^)	0.245 ± 0.115	0.280 ± 0.120	0.484
Perimeter (mean ± SD, mm)	2.269 ± 0.564	2.176 ± 0.581	**0.018**
Circularity (mean ± SD)	0.652 ± 0.088	0.704 ± 0.098	**<0.001**

SD, standard deviation. The *p* value was obtained using a paired *t*-test. Boldface numbers indicate statistically significant differences at *p* < 0.05.

**Table 3:** Repeatability of vascular density, perfusion density, and foveal avascular zone metrics according to measurement areas.

	ICC		CV		CR	
	3 × 3 mm	6 × 6 mm	3 × 3 mm	6 × 6 mm	3 × 3 mm	6 × 6 mm
Vessel density						
Total area	0.812 (0.732–0.867)	0.816 (0.739–0.871)	3.92 (3.25–4.59)	3.70 (2.88–4.53)	2.11 (1.99–2.23)	1.69 (1.57–1.81)
Fovea	0.826 (0.744–0.878)	0.741 (0.693–0.812)	8.00 (6.73–9.28)	12.75 (10.75–14.75)	2.37 (2.21–2.52)	2.70 (2.57–2.82)
Inner average	0.812 (0.733–0.868)	0.734 (0.622–0.812)	3.68 (3.06–4.31)	5.66 (4.23–7.08)	2.11 (1.99–2.34)	2.47 (2.30–2.64)

Perfusion density						
Total area	0.823 (0.748–0.875)	0.832 (0.761–0.881)	3.57 (2.98–4.16)	3.81 (2.93–4.70)	0.03 (0.03–0.04)	0.04 (0.04–0.05)
Fovea	0.839 (0.761–0.886)	0.762 (0.685–0.783)	9.32 (7.90–10.74)	14.90 (12.41–17.40)	0.05 (0.04–0.05)	0.08 (0.07–0.08)
Inner average	0.803 (0.720–0.861)	0.760 (0.659–0.831)	3.41 (2.82–4.01)	5.96 (4.46–7.47)	0.04 (0.03–0.04)	0.06 (0.06–0.07)

Foveal avascular zone						
Area	0.892 (0.847–0.924)	0.695 (0.630–0.817)	4.65 (3.56–5.73)	12.75 (10.09–15.41)	0.06 (0.05–0.07)	0.13 (0.12–0.14)
Perimeter	0.773 (0.607–0.875)	0.694 (0.566–0.785)	6.45 (3.98–8.92)	11.87 (8.81–14.93)	0.55 (0.48–0.61)	0.69 (0.63–0.76)
Circularity	0.757 (0.578–0.846)	0.503 (0.294–0.650)	8.53 (6.90–10.16)	10.99 (8.34–13.63)	0.17 (0.16–0.19)	0.20 (0.18–0.21)

ICC: intraclass correlation; CV: coefficient of variation; CR: coefficient of repeatability. Repeatability indicators are presented with 95% confidence intervals.

## Data Availability

The data used to support the findings of this study are available from the corresponding author upon request.

## Supplementary Materials

Supplementary Materials Supplementary material 1: comparison of vascular density and perfusion density according to the Early Treatment of Diabetic Retinopathy Study between 3 × 3 mm and 6 × 6 mm scans. Supplementary material 2: repeatability of vascular density and perfusion density according to the Early Treatment of Diabetic Retinopathy Study.
