# Supplementary material for: The Difference in Repeatability of Automated Superficial Retinal Vessel Density according to the Measurement Area Using OCT Angiography
Source: J Ophthalmol. 2020 Apr 17;2020:5686894. doi: 10.1155/2020/5686894 (PMC7183530; doi:10.1155/2020/5686894)
Supplement: Supplementary Materials — Supplementary material 1: comparison of vascular density and perfusion density according to the Early Treatment of Diabetic Retinopathy Study between 3 × 3 mm and 6 × 6 mm scans. Supplementary material 2: repeatability of vascular density and perfusion density according to the Early Treatment of Diabetic Retinopathy Study. [file 5686894.f1.docx]

Supplementary material 1. Comparison of vascular density and perfusion density according to the Early Treatment of Diabetic Retinopathy Study between 3×3 mm and 6×6 mm scans

|  | **3×3 mm scan** | **6×6 mm scan** | **p-value** |
| --- | --- | --- | --- |
| **Vessel density (mean ± SD, mm^-1^)** |  |  |  |
| **InnerAverage** | 22.06 ± 1.75 | 18.65 ± 0.92 | **<0.001** |
| **InnerSuperior** | 21.28 ± 1.10 | 17.37 ± 1.03 | **<0.001** |
| **InnerNasal** | 21.53 ± 0.91 | 17.86 ± 0.95 | **<0.001** |
| **InnerInferior** | 21.10 ± 0.95 | 17.71 ± 0.96 | **<0.001** |
| **InnerTemporal** | 21.12 ± 1.02 | 17.51 ± 1.11 | **<0.001** |
| **OuterAverage** |  | 18.83 ± 0.68 |  |
| **OuterSuperior** |  | 18.13 ± 0.71 |  |
| **OuterNasal** |  | 19.63 ± 0.44 |  |
| **OuterInferior** |  | 18.06 ± 0.58 |  |
| **OuterTemporal** |  | 16.42 ± 1.11 |  |
| **Perfusion density (mean ± SD)** |  |  |  |
| **InnerAverage** | 0.382 ± 0.013 | 0.422 ± 0.022 | **<0.001** |
| **InnerSuperior** | 0.383 ± 0.021 | 0.426 ± 0.025 | **<0.001** |
| **InnerNasal** | 0.386 ± 0.018 | 0.421 ± 0.024 | **<0.001** |
| **InnerInferior** | 0.380 ± 0.017 | 0.426 ± 0.024 | **<0.001** |
| **InnerTemporal** | 0.382 ± 0.020 | 0.414 ± 0.028 | **<0.001** |
| **OuterAverage** |  | 0.447 ± 0.014 |  |
| **OuterSuperior** |  | 0.453 ± 0.019 |  |
| **OuterNasal** |  | 0.482 ± 0.011 |  |
| **OuterInferior** |  | 0.448 ± 0.015 |  |
| **OuterTemporal** |  | 0.404 ± 0.028 |  |

SD, standard deviation.

The p-value was obtained using a paired *t*-test.

Boldface numbers indicate statistically significant differences at p < 0.05.

Supplementary material 2. Repeatability of vascular density and perfusion density according to the Early Treatment of Diabetic Retinopathy Study

|  | **ICC** | | **CV** | | **CR** | |
| --- | --- | --- | --- | --- | --- | --- |
|  | 3×3 mm | 6×6 mm | 3×3 mm | 6×6 mm | 3×3 mm | 6×6 mm |
| **Vessel density** |  |  |  |  |  |  |
| **InnerAverage** | 0.812  (0.733–0.868) | 0.734  (0.622–0.812) | 3.68  (3.06–4.31) | 5.66  (4.23–7.08) | 2.11  (1.99–2.34) | 2.47  (2.30–2.64) |
| **InnerSuperior** | 0.752  (0.646–0.835) | 0.604  (0.437–0.721) | 5.43  (4.30–6.57) | 6.57  (4.64–8.50) | 3.01  (2.85–3.26) | 2.85  (2.62–3.07) |
| **InnerNasal** | 0.821  (0.746–0.874) | 0.740  (0.645–0.824) | 4.40  (3.66–5.13) | 5.92  (4.55–7.30) | 2.51  (2.37–2.65) | 2.64  (2.45–2.84) |
| **InnerInferior** | 0.776  (0.682–0.842) | 0.697  (0.570–0.787) | 4.67  (3.86–5.50) | 6.23  (4.50–7.95) | 2.62  (2.47–2.77) | 2.66  (2.47–2.84) |
| **InnerTemporal** | 0.756  (0.653–0.828) | 0.701  (0.576–0.790) | 5.08  (4.20–5.97) | 7.24  (5.62–8.85) | 2.83  (2.67–3.00) | 3.08  (2.87–3.29) |
| **OuterAverage** |  | 0.828  (0.755–0.878) |  | 3.29  (2.56–4.02) |  | 1.55  (1.43–1.66) |
| **OuterSuperior** |  | 0.772  (0.676–0.839) |  | 4.24  (3.25–5.22) |  | 1.98  (1.83–2.12) |
| **OuterNasal** |  | 0.784  (0.693–0.848) |  | 2.36  (1.76–2.96) |  | 1.22  (1.13–1.33) |
| **OuterInferior** |  | 0.855  (0.793–0.898) |  | 3.45  (2.68–4.22) |  | 1.60  (1.49–1.71) |
| **OuterTemporal** |  | 0.753  (0.650–0.826) |  | 7.81  (5.93–9.70) |  | 3.07  (2.84–3.30) |
| **Perfusion density** |  |  |  |  |  |  |
| **InnerAverage** | 0.803  (0.720–0.861) | 0.760  (0.659–0.831) | 3.41  (2.82–4.01) | 5.96  (4.46–7.47) | 0.04  (0.03–0.04) | 0.06  (0.06–0.07) |
| **InnerSuperior** | 0.782  (0.695–0.850) | 0.643  (0.493–0.749) | 5.61  (4.40–6.82) | 6.79  (4.77–8.81) | 0.06  (0.05–0.60) | 0.07  (0.06–0.08) |
| **InnerNasal** | 0.762  (0.662–0.832) | 0.755  (0.652–0.827) | 4.76  (3.92–5.60) | 6.33  (4.84–7.83) | 0.05  (0.04–0.05) | 0.07  (0.06–0.07) |
| **InnerInferior** | 0.758  (0.657–0.830) | 0.730  (0.617–0.810) | 4.72  (3.89–5.55) | 6.48  (4.68–8.27) | 0.05  (0.04–0.05) | 0.07  (0.06–0.07) |
| **InnerTemporal** | 0.771  (0.683–0.843) | 0.718  (0.599–0.801) | 5.53  (4.53–6.52) | 7.93  (6.18–9.69) | 0.06  (0.05–0.06) | 0.08  (0.07–0.08) |
| **OuterAverage** |  | 0.838  (0.769–0.886) |  | 3.41  (2.61–4.21) |  | 0.05  (0.05–0.06) |
| **OuterSuperior** |  | 0.770  (0.674–0.838) |  | 4.51  (3.42–5.60) |  | 0.05  (0.05–0.06) |
| **OuterNasal** |  | 0.807  (0.725–0.864) |  | 2.49  (1.83–3.16) |  | 0.03  (0.03–0.04) |
| **OuterInferior** |  | 0.855  (0.795–0.898) |  | 3.61  (2.79–4.43) |  | 0.04  (0.03–0.04) |
| **OuterTemporal** |  | 0.785  (0.695–0.849) |  | 8.09  (6.26–9.92) |  | 0.08  (0.07–0.08) |

ICC, intraclass correlation; CV, coefficient of variation; CR, coefficient of repeatability.

Repeatability indicators are presented with 95% confidence intervals.
